# *Mycobacterium tuberculosis *septum site determining protein, Ssd encoded by *rv3660c*, promotes filamentation and elicits an alternative metabolic and dormancy stress response

**DOI:** 10.1186/1471-2180-11-79

**Published:** 2011-04-19

**Authors:** Kathleen England, Rebecca Crew, Richard A Slayden

**Affiliations:** 1 Mycobacteria Research Laboratories, Department of Microbiology, Immunology, and Pathology. Colorado State University, Fort Collins, CO 80523, USA; 2 Tuberculosis Research Section, NIH/NIAID, 9000 Rockville Pike, Bldg 33, Room 2W20D, Bethesda, Maryland, 20892-3206, USA

**Keywords:** *Mycobacterium tuberculosis*, dormancy, Dos regulon, septum site determining protein, cell division

## Abstract

**Background:**

Proteins that are involved in regulation of cell division and cell cycle progression remain undefined in *Mycobacterium tuberculosis*. In addition, there is a growing appreciation that regulation of cell replication at the point of division is important in establishing a non-replicating persistent state. Accordingly, the objective of this study was to use a systematic approach consisting of consensus-modeling bioinformatics, ultrastructural analysis, and transcriptional mapping to identify septum regulatory proteins that participate in adaptive metabolic responses in *M. tuberculosis*.

**Results:**

Septum site determining protein (Ssd), encoded by *rv3660c *was discovered to be an ortholog of septum site regulating proteins in actinobacteria by bioinformatics analysis. Increased expression of *ssd *in *M. smegmatis *and *M. tuberculosis *inhibited septum formation resulting in elongated cells devoid of septa. Transcriptional mapping in *M. tuberculosis *showed that increased *ssd *expression elicited a unique response including the dormancy regulon and alternative sigma factors that are thought to play a role in adaptive metabolism. Disruption of *rv3660c *by transposon insertion negated the unique transcriptional response and led to a reduced bacterial length.

**Conclusions:**

This study establishes the first connection between a septum regulatory protein and induction of alternative metabolism consisting of alternative sigma factors and the dormancy regulon that is associated with establishing a non-replicating persistent intracellular lifestyle. The identification of a regulatory component involved in cell cycle regulation linked to the dormancy response, whether directly or indirectly, provides a foundation for additional studies and furthers our understanding of the complex mechanisms involved in establishing a non-replicating state and resumption of growth.

## Background

Despite effective chemotherapeutic regimens, *Mycobacterium tuberculosis *remains one of the most significant public health problems, with an estimated global burden of one third of the world's population. The unremitting global burden is attributed, in part, to the ability of *M. tuberculosis *to establish and maintain a non-replicating persistent infection, thus making the bacillus tolerant to drug treatment and host immune response [1,2]. Studies have demonstrated that the development of non-replicating persistence involves a shift from rapid to slow growth followed by a complete shutdown of cell cycle progression characterized by a complete round of DNA replication and inhibition of cell division [3-5]. These experimental observations indicate that cell division, and septum formation in particular, is a key regulatory checkpoint of the cell cycle for entry into a non-replicating state. However, proteins that regulate septum formation as part of growth arrest and altered metabolic responses associated with the persistent state remain undefined in *M. tuberculosis*. Thus, it is important to identify regulatory elements involved in septum formation and the cell cycle in context of adaptive metabolism and to the development of a non-replicating persistent state.

Cell cycle progression in bacteria, including *M. tuberculosis*, is governed in response to stress conditions substantiating the notion that septum regulation and cell division events are regulated under a variety of circumstances [6-10]. Response and adaption to stress is a complex series of events that relies on coordination of multiple processes. The prototypical stress response is the SOS response, which involves check-point regulation and de-repression of genes under direct and indirect control of a common repressor. Eliciting the SOS response leads to a cessation in cell division due to inhibition of FtsZ polymerization via SulA, and transient induction of alternative functions [11,12]. In addition to DNA repair, there are other mechanisms that are controlled by the SOS response, thus establishing that responses to stress share common components with regards to regulation. Similarly, in *M. tuberculosis *inhibition of FtsZ polymerization and cell division occurs in response to stress conditions, which include environmental changes that occur during pathogenesis and drug treatment. Therefore, inhibition of septum formation through the regulation of FtsZ polymerization represents a common mechanism that is conserved among bacteria, including *M. tuberculosis*, to control cell division and cell cycle activity in response to various conditions including stress [8].

In model organisms, FtsZ polymerization is controlled under normal growth conditions by a variety of FtsZ interacting regulatory elements including *Min*-system proteins, Div proteins, MipZ and under stress conditions by proteins such as SulA [13]. In Gram-negative organisms septum site selection and regulation are controlled by the *Min*-system consisting of MinC, MinD and MinE, while in Gram-positive organisms the system consists of MinC, MinD, and an ortholog DivIVa. Along with these proteins, other proteins that have a demonstrated regulation in FtsZ polymerization have been identified; however the precise role these regulatory components play is not well defined. One group of FtsZ regulatory proteins is the septum site determining proteins. This family of proteins has limited similarity to proteins involved in morphological differentiation in Streptomyces spp. These components work together to negatively regulate FtsZ polymerization preventing cell division until DNA replication is complete and the chromosomes have been properly segregated.

It is well accepted that during establishment of a chronic latent infection *M. tuberculosis *halts cell cycle progression and significantly reduces metabolic activity. One adaptive process that has been associated with limited growth conditions, stress, and pathogenesis is the *Dos-*response. Under experimental conditions, the Dos regulon is induced in response hypoxia, NO and carbon monoxide [14]. The *Dos-*response is generally thought to be important for adaptation to alternative growth conditions, thus establishing the ability to endure long periods within the host. The idea that the *Dos-*response plays a role in pathogenesis is supported by studies that have demonstrated that the highly virulent W-Beijing linage of *M. tuberculosis *exhibits high levels of constitutive expression of the *Dos-*regulon components [15,16]. While the DosR two-component regulatory system and primary members of the *Dos-*regulon are well defined, other components, particularly complimentary regulatory elements that coordinate cell cycle progression and growth in response to alternative growth conditions remain undefined. Because bioinformatics approaches alone have failed to identify homologs for all cell cycle components, we have previously used inhibition of cell division and transcriptional mapping to identify putative regulatory elements in *M. tuberculosis*, with particular focus on those that regulate septum formation [6,7,17].

The detailed regulatory mechanisms involved in inhibition of septum formation and cell division in *M. tuberculosis *have not been defined, and will afford an understanding of the mechanisms involved with growth and adaptation to alternative environments signaling the induction of bacteria into a non-replicating state. In order to identify septum regulatory proteins that elicit a transcriptional stress response, a systematic approach consisting of consensus-modeling bioinformatics, gene dosage and ultrastructural analysis, and expression profiling was employed. As a result, *rv3660c *was discovered to encode a protein with similarity to the loosely defined family of septum site determining proteins. Increased expression of *rv3360c *resulted in filamentous cells, while the disruption of the gene by transposon insertion presented minicell morphology demonstrating an inhibitory role in septum formation. Transcriptional analysis showed that *rv3660c *expression results in the induction of a unique profile of alternative sigma factors, open reading frames encoding proteins involved in alternative metabolism and the dormancy regulon. Accordingly, this is the first report of a Ssd-like septum regulating protein in *M. tuberculosis*, and that stalls cell division and is associated with induction of alternative metabolism associated with pathogenesis and survival of non-replicating bacilli, thus representing a previously unidentified regulatory mechanism in *M. tuberculosis*. These data, in combination with previous studies to identify septum regulatory elements in *M. tuberculosis*, indicate that the protein encoded by *rv3360c *is Ssd, a septum site determining protein.

## Results

### *rv3660c *encodes a previously unidentified septum site determining-like protein, Ssd

**Figure 1:**
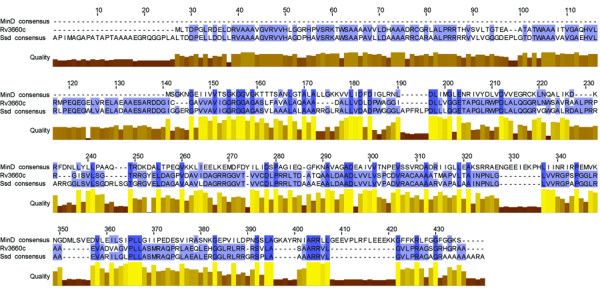
**Protein alignments**. Alignment of MinD protein consensus sequence, septum site determining (Ssd) protein consensus sequence and the *M. tuberculosis *Ssd protein encoded by (*rv3660c*). The MinD proteins consensus was from OMA Group 78690 and septum site determining proteins consensus was from OMA Group 73337. The protein conservation, quality and overall consensus for the alignments are indicated.

A bioinformatics approach utilizing consensus sequences derived from global alignments of annotated MinD proteins (OMA Group 78690) and septum site determining proteins (OMA Group 73337) was taken to search the *M. tuberculosis *H37Rv genome for open reading frames that encode putative *MinD*-like and *Ssd*-like orthologs. The search using the Ssd consensus identified the conserved hypothetical open reading frame *rv3660c*, which is consistent with previous bioinformatics and experimental assignment. Search of the *M. tuberculosis *genome with the MinD consensus sequence also identified *rv3660c*, but with less similarity to MinD orthologs with 30% sequence similarity. Identification of Rv3660c using both Ssd and MinD consensus models strongly indicates that *rv3660c *encodes a FtsZ regulatory protein. Alignments of the protein encoded by *rv3660c *with the MinD and Ssd consensus sequences confirmed and substantiated that the protein encoded by *rv3660c *is a member of the septum site determining protein family (Figure 1). Further evidence that *rv3660c *encoded a Ssd protein was obtained from hierarchical clustering analysis of Ssd encoded by *rv3660c*, 46 proteins annotated as MinD and 37 proteins annotated as Ssd. Hierarchical clustering analysis resulted in SsD (Rv3660c) grouping with Ssd proteins encoded in actinobacteria. This data is consistent with previous data that, *rv3660c *was mapped to septum formation in transcriptional mapping studies [6].

### *ssd *expression promotes filamentation in *M. smegmatis *and *M. tuberculosis*

**Figure 2:**
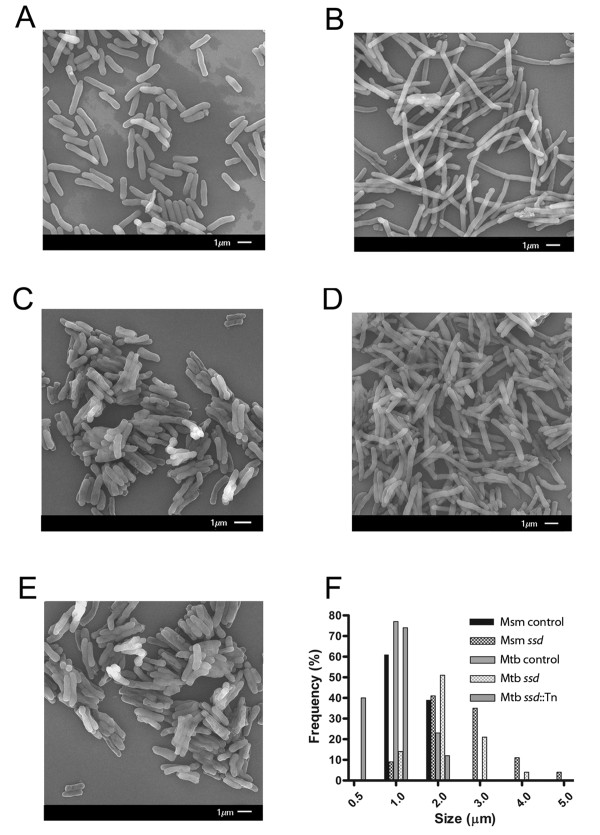
**Ultrastructure Analysis (SEM) and Length distributions**. Bacterial morphology. (A) *M. smegmatis *control strain, (B) *M. smegmatis ssd *merodiploid (C) *M. tuberculosis *control, (D) *M. tuberculosis ssd *merodiploid and (E) *ssd*::Tn mutant *M. tuberculosis *strain were visualized by scanning electron microscopy. Images are representative of different fields of bacteria from exponentially growing cultures at 37°C. (F) Lengths of the bacterial cells were calculated from the coordinates of both ends of the cell as measured from representative fields as visualized by scanning electron microscopy. Multiple fields were examined and values calculated in 0.5-1 mm increments from multiple fields of over 100 cells.

To assess if Ssd inhibits septum formation in mycobacteria, gene dosage studies were conducted in *M. smegmatis *and *M. tuberculosis*, and bacterial ultrastructure was visualized and measured by scanning electron microscopy (Figure 2). The expression of *ssd *in merodiploid strains was assessed by quantitative RT-PCR and production was confirmed by western blot analysis. Expression of *ssd *was more robust in *M. smegmatis *than *M. tuberculosis *as compared to SigA expression. In the *M. tuberculosis *merodiploid strain *ssd *expression was 10-20 fold increased on average over endogenous expression levels. These studies also revealed that *ssd *is constitutively expressed at low levels throughout the growth cycle under laboratory growth conditions. This observation is consistent with the oberserved low level expression of other stress responses [14,16]. There was no significant difference in the growth rate or physical characteristics, such as clumping or pigmentation between *M. smegmatis *and *M. tuberculosis *strains expressing *ssd *and control strains. The primary distinguishing physical feature between the *M. smegmatis *and *M. tuberculosis ssd *expressing merodiploid strains in comparison to control bacteria was increased cell lengths and a smooth ultrastructural characteristic (Figure 2ABCD). The observed smooth ultrastructure devoid of concentric rings along the bacterial filament is important because this observation is consistent with inhibition of FtsZ polymerization and Z-ring formation as previously reported [6,7,17,18]. The *M. smegmatis *wild type control strain exhibited cell lengths of 2.1 ± 0.11 μm (Figure 2AF) and the *M. smegmatis ssd *merodiploid strain had increased cell lengths of 3.2 ± 0.42 μm (Figure 2BF). Similarly, *M. tuberculosis *H37Rv control cells had lengths of 1.73 ± 0.43 μm (Figure 2CF) and expression of *ssd *resulted in increased cell lengths of 2.53 ± 0.76 μm (Figure 2DF). In contrast, a *ssd*::Tn *M. tuberculosis *mutant strain had decreased cell lengths of 1.35 ± 0.51 μm (Figure 2EF). This experimental data demonstrates a causal relationship between the expression levels of *ssd *and altered bacterial cell lengths, confirming the bioinformatics analysis and further substantiating Ssd as a septum regulation protein as annotated (http://genolist.pasteur.fr/TubercuList[19]) and indicated by transcriptional mapping [6].

### Whole-genome expression profiling of *ssd *merodiploid and mutant strains

To assess the effect of *ssd *expression on *M. tuberculosis *metabolism, global gene expression profiling was performed on the *ssd *overexpression *M. tuberculosis *merodiploid strain. A total of 2,274 ORFs were transcriptionally active with 432 of these ORFs being differentially expressed 1.5-fold or greater change (p values ≤ 0.05). Overall, genes with altered transcription encode proteins involved in lipid metabolism, cell respiration, protein synthesis, cell wall surface molecules, cell cycle progression, and most notably genes involved in dormancy and stress.

The genes induced to the greatest extent as a result of increased *ssd *expression were alternative sigma factors and members of the *dosR*-regulon and (Table 1). The *dosR*-dependent genes (*rv3131*, *hspX *and *tgs1) *and the alternative sigma factors (*sigF, sigG, sigH sigI, sigJ, sigL and sigM) *along with genes involved in adaptive metabolic functions such as anaerobic respiration (*frdAB*, *nirBD, narI, narJ, narG, narU, narX and narK2)*, electron transport and redox-potential (*ackA, fprB, cydC, cydB, appC, fdxA, and rubA*), and genes associated with fatty acid degradation (*fad, ech, acc, mut*) were induced. In additional to the increased expression of genes involved in adaptive metabolism and stress, the *ssd *merodiploid induced the expression of polyketide genes *pks6-11, 17 and 18 *and various lipoprotein genes *lpp and lpq *(Table 2). These genes are also associated with adaptive responses to alternative growth conditions and have been shown to contribute to virulence traits in *M. tuberculosis *[20]. In contrast, genes encoding ribosomal proteins (*rpl, rps, rpm*) required for protein synthesis were downregulated. These transcriptional activities are concordant with increased transcriptional activity of genes involved in dormancy, adaptive responses, and conditions associated with a non-replicating persistent lifestyle.

**Table 1:** *dosR *regulon gene expression from transcriptional profiles of *ssd *merodiploid strain and the *ssd*::Tn mutant strain

Locus	Gene	Product	*merodiploid*		*mutant*		Δ
			***Log***_***2 ***_***exp***	***p-value***	***Log***_***2 ***_***exp***	***p-value***	

Rv0079		hypothetical protein	1.31	0.007	0.27	0.000	4.9

Rv0080		hypothetical protein	1.35	0.002	0.20	0.001	6.7

Rv0081		transcriptional regulator (ArsR family)	1.10	0.000	0.20	0.016	5.4

Rv0082		probable oxidoreductase subunit	0.46	0.011	0.28	0.063	1.7

Rv0083		probable oxidoreductase subunit	0.10	0.001	0.88	0.008	0.1

Rv0569		conserved hypothetical protein	1.26	0.000	0.29	0.003	4.3

Rv0570	*nrdZ*	ribonucleotide reductase, class II	1.19	0.018	-0.08	0.003	-15.0

Rv0571c		conserved hypothetical protein	0.14	0.025	-0.15	0.000	-0.9

Rv0572c		hypothetical protein	0.30	0.002	-0.41	0.013	-0.7

Rv0573c		conserved hypothetical protein	0.83	0.006	0.19	0.000	4.4

Rv0574c		conserved hypothetical protein	0.76	0.009	-0.23	0.006	-3.2

Rv1733c		possible membrane protein	1.99	0.068	0.33	0.002	6.0

Rv1734c		hypothetical protein	0.71	0.013	-0.04	0.009	-18.0

Rv1735c		hypothetical protein	0.50	0.001	0.14	0.012	3.4

Rv1736c	*narX*	fused nitrate reductase	1.09	0.032	0.07	0.000	15.0

Rv1737c	*narK2*	nitrite extrusion protein	1.87	0.228	0.20	0.001	9.2

Rv1738		conserved hypothetical protein	2.90	0.230	0.96	0.016	3.0

Rv1812c		probable dehydrogenase	0.03	0.324	-0.15	0.001	-0.2

Rv1813c		conserved hypothetical protein	1.26	0.257	1.83	0.030	0.7

Rv1996		conserved hypothetical protein	2.63	0.046	0.80	0.025	3.3

Rv1997	*ctpF*	probable cation transport ATPase	1.62	0.001	0.17	0.018	9.4

Rv1998c		conserved hypothetical protein	0.47	0.118	0.10	0.000	4.6

Rv2003c		conserved hypothetical protein	1.26	0.004	0.08	0.010	15.1

Rv2004c		hypothetical protein	1.01	0.008	0.36	0.022	2.8

Rv2005c		conserved hypothetical protein	1.78	0.033	0.33	0.000	5.4

Rv2006	*otsB2*	trehalose-6-phosphate phosphatase	1.28	0.000	0.02	0.008	78.4

Rv2007c	*fdxA*	ferredoxin	2.56	0.137	0.64	0.026	4.0

Rv2027c	*dosT*	sensor histidine kinase	1.35	0.001	0.07	0.044	18.9

Rv2028c		conserved hypothetical protein	0.38	0.009	-0.11	0.004	-3.3

Rv2029c	*pfkB*	phosphofructokinase II	2.03	0.330	0.26	0.006	7.8

Rv2030c		conserved hypothetical protein	3.37	0.195	0.62	0.004	5.4

Rv2031c	*hspX*	14 kD antigen, heat shock protein Hsp20 family	3.94	0.043	1.50	0.079	2.6

Rv2032	*acg*	conserved hypothetical protein	2.50	0.277	0.29	0.003	8.6

Rv2617c		hypothetical protein	-0.21	0.012	-0.01	0.000	20.6

Rv2623		conserved hypothetical protein	3.02	0.151	0.15	0.132	19.8

Rv2624c		conserved hypothetical protein	1.34	0.062	0.10	0.024	13.9

Rv2625c		conserved hypothetical protein	-0.03	0.016	-0.94	0.017	0.0

Rv2626c		conserved hypothetical protein	3.35	0.000	0.77	0.184	4.4

Rv2627c		conserved hypothetical protein	2.65	0.285	0.05	0.010	51.0

Rv2628		hypothetical protein	2.22	0.022	0.14	0.038	16.0

Rv2629		hypothetical protein	0.49	0.004	0.28	0.006	1.8

Rv2630		hypothetical protein	1.42	0.003	0.24	0.014	5.9

Rv2631		conserved hypothetical protein	0.70	0.015	-0.17	0.021	-4.1

Rv2830c		similar to phage P1 phd gene	0.29	0.000	-0.07	0.002	-3.9

Rv3126c		hypothetical protein	0.91	0.021	0.07	0.018	12.8

Rv3127		conserved hypothetical protein	2.15	0.044	0.51	0.000	4.2

Rv3128c		conserved hypothetical protein	0.30	0.310	0.13	0.002	2.3

Rv3129		conserved hypothetical protein	1.09	0.002	0.03	0.035	40.6

Rv3130c	*tgs1*	conserved hypothetical protein	3.92	0.309	0.84	0.013	4.7

Rv3131		conserved hypothetical protein	4.01	0.273	1.66	0.189	2.4

Rv3132c	*dosS*	sensor histidine kinase	2.00	0.014	0.18	0.001	11.0

Rv3133c	*dosR*	two-component response regulator	1.00	0.070	0.22	0.009	4.5

Rv3134c		conserved hypothetical protein	2.45	0.024	0.16	0.002	15.0

Rv3841	*bfrB*	bacterioferritin	1.22	0.106	1.36	0.087	0.9

**Table 2:** Genes differentially regulated for selected cell functions (p-value ≤ 0.05)

ORF	Gene	**Log **_**2 **_**expression**		ORF	Gene	**Log **_**2 **_**expression**	
		***merodiploid***	***mutant***			***merodiploid***	***mutant***

***Fatty acid utilization***				***Ribosomal proteins***			

Rv0974c	*accD2*	1.2	-0.2	Rv0056	*rplI*	-1.0	-0.6

Rv1935c	*echA13*	0.9	-0.2	Rv0682	*rpsL*	-0.9	-0.9

Rv2486	*echA14*	1.0	-0.1	Rv0700	*rpsJ*	-1.4	-0.5

Rv0456c	*echA2*	1.2	-0.1	Rv0701	*rplC*	-1.5	-0.4

Rv3550	*echA20*	1.1	0.2	Rv0716	*rplE*	-1.2	-0.9

Rv0971c	*echA7*	1.3	-0.1	Rv0722	*rpmD*	-0.9	-0.3

Rv3546	*fadA5*	1.1	0.1	Rv0723	*rplO*	-0.7	-0.2

Rv1715	*fadB3*	1.0	-0.1	Rv2441c	*rpmA*	-0.9	-0.5

Rv0099	*fadD10*	1.2	0.0	Rv3442c	*rpsI*	-0.9	-0.2

Rv1550	*fadD11*	1.0	0.2	Rv3443c	*rplM*	-1.6	-0.5

Rv1058	*fadD14*	1.2	0.0	Rv3458c	*rpsD*	-0.8	-0.5

Rv3561	*fadD3*	0.8	0.5	Rv3460c	*rpsM*	-1.3	-0.6

Rv0035	*fadD34*	1.3	0.0	Rv3461c	*rpmJ*	-1.4	-0.6

Rv0214	*fadD4*	0.8	-0.2	Rv3924c	*rpmH*	-1.2	-0.7

Rv0551c	*fadD8*	0.9	0.0				

Rv2590	*fadD9*	1.3	-0.5	***Anaerobic respiration***			

Rv0972c	*fadE12*	1.4	-0.1	Rv0252	*nirB*	0.8	ndr

Rv0975c	*fadE13*	1.3	ndr	Rv0253	*nirD*	1.1	ndr

Rv3061c	*fadE22*	1.0	-0.1	Rv0267	*narU*	1.2	ndr

Rv3505	*fadE27*	1.0	0.0	Rv1161	*narG*	0.7	ndr

Rv3544c	*fadE28*	0.8	0.0	Rv1163	*narJ*	0.7	ndr

Rv3562	*fadE31*	1.0	0.2	Rv1164	*narI*	0.6	ndr

Rv3563	*fadE32*	0.8	0.4	Rv1552	*frdA*	1.2	ndr

Rv3564	*fadE33*	1.2	0.3	Rv1553	*frdB*	0.8	ndr

Rv0752c	*fadE9*	0.9	-0.1	Rv1554	*frdC*	1.1	ndr

Rv1492	*mutA*	1.1	0.2	Rv1736c	*narX*	1.1	ndr

Rv1493	*mutB*	1.2	0.5	Rv1737c	*narK2*	1.9	0.2

							

***Cell surface molecules***				***Electron Trpt/Redox***			

Rv0399c	*lpqK*	0.8	-0.1	Rv0409	*ackA*	1.0	0.2

Rv0405	*pks6*	1.2	-0.2	Rv0886	*fprB*	0.8	0.1

Rv0593	*lprL*	1.1	0.0	Rv1620c	*cydC*	1.6	0.0

Rv0604	*lpqO*	0.8	-0.1	Rv1622c	*cydB*	2.0	-0.2

Rv0794c	*lpdB*	0.9	-0.2	Rv1623c	*appC*	1.0	-0.2

Rv1064c	*lpqV*	1.1	-0.1	Rv2007c	*fdxA*	2.6	0.6

Rv1166	*lpqW*	0.8	0.0	Rv3251c	*rubA*	0.8	-0.1

Rv1372	*pks18*	1.1	0.1				

Rv1661	*pks7*	1.3	-0.2	***ATP synthesis***			

Rv1662	*pks8*	1.0	0.2	Rv1304	*atpB*	0.2	-0.6

Rv1663	*pks17*	1.2	0.2	Rv1305	*atpE*	0.2	-0.4

Rv1664	*pks9*	1.1	0.1	Rv1306	*atpF*	0.0	-0.7

Rv1665	*pks11*	0.7	0.2	Rv1307	*atpH*	0.2	-0.6

Rv1921c	*lppF*	1.4	0.2	Rv1308	*atpA*	0.3	-0.4

Rv1946c	*lppG*	1.0	0.1	Rv1309	*atpG*	-0.1	-0.7

Rv1966	*mce3*	1.1	0.0	Rv1310	*atpD*	0.3	-0.4

Rv2270	*lppN*	0.9	-0.1	Rv1311	*atpC*	0.2	-0.4

Rv2330c	*lppP*	0.7	0.1				

Rv2543	*lppA*	0.9	0.2	*ndr = not differentially regulated*			

Rv2796c	*lppV*	0.8	0.0				

To determine whether the observed *dos*-response was a direct result of *ssd *expression, transcriptional analysis of the *ssd*::Tn mutant *M. tuberculosis *strain was performed. Compared to the *ssd *merodiploid strain, only 65 genes displayed a 1.5-fold or greater (p values ≤ 0.05) change in expression in the *ssd *mutant. Of notable absence in the transcriptional response in the *ssd*::Tn mutant strain are genes of the *dos*-regulon the other stress associated genes, and the virulence-associated genes that were identified in the *ssd *overexpressing mutant strain. The observed limited number of differentially expressed genes includes those involved in the cell cycle processes of lipid biosynthesis *(kasA and kasB*), the chromosome partitioning gene *parA*, and the *divIVa *homologue, *wag31*. Notably, *parA*, and the *divIVa *are known to be involved in regulation and coordination of chromosome partitioning and septum placement events, which is consistent with a mild disruption in coordination of chromosome partitioning and cell division. Thus, the contrasting and unique induction of the *dos-*regulon, alternative sigma factors and virulence genes upon *ssd *overexpression indicates that these responses result from increased levels of *ssd *and are connected to regulatory events involved in septum formation.

**Figure 3:**
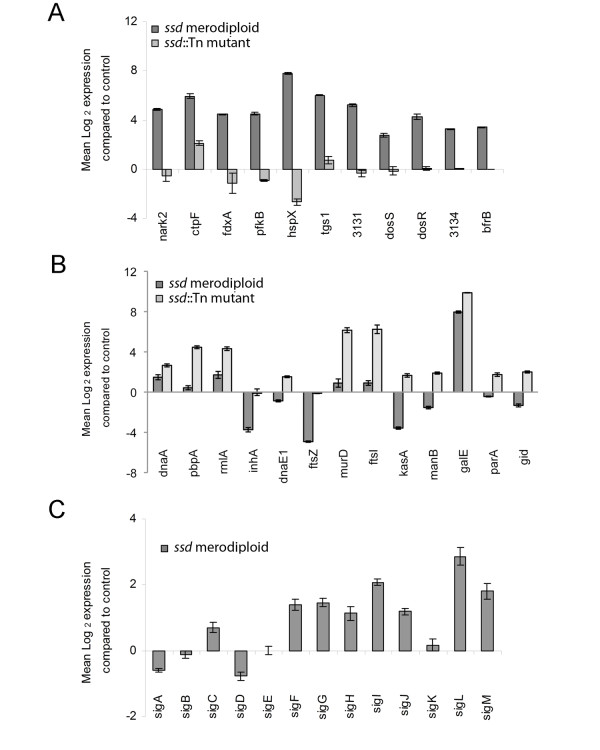
**Quantitative real time-PCR analysis of select genes**. Mean log_2 _expression for (A) representative *dosR *regulon genes, (B) cell cycle discriminant genes and (C) sigma factors in the *ssd *merodiploid *M. tuberculosis *strain compared to *M. tuberculosis *control strain. Data are mean values ± SD from independent biological samples. Ratios were calculated using the total number of gene targets from the *ssd *merodiploid *M. tuberculosis *strain or *ssd*::Tn mutant *M. tuberculosis *strain compared to paired *M. tuberculosis *control stain.

The differentially expressed *dos*-regulated genes, cell cycle discriminant genes and sigma factors identified by microarray were validated by quantitative RT-PCR analysis (Figure 3). The concordance in expression trends of these genes as determined by microarray and quantitative RT-PCR specifically verify that *ssd *expression induced genes of the *dos*-regulon and stress genes (Figure 3A), with altered expression of cell cycle genes (Figure 3B), all of which are consistent with septum inhibition. With regards to the sigma factors, *sigA *expression was repressed in the *ssd *merdodiploid strain while the alternative sigma factors *sigF, sigG, sigH. sigI, sigJ, sigL *and *sigM *were induced (Figure 3C). The quantitative RT-PCR analysis was concordant with the expression trends observed by microarray and confirmed that *ssd *expression elicits a *dosR-*like stress response consisting of known *dos-*members and alternative sigma factors, which was not observed in the *ssd *mutant.

## Discussion

*M. tuberculosis *is able to circumvent host responses and establish a latent infection where it can silently persist for years. While the bacterial response to growth in various environments has been reported, the proteins that participate in the complex regulatory processes that govern growth in response to stress or changing environments remain largely unknown. Proteins that are orthologs of know septum formation regulatory elements are candidates for participating in non-replicating persistence because the reversible "off" and "on" regulation allows relapse of disease. Accordingly, a consensus sequence modeling approach was employed to identify putative septum formation inhibitors and, genes dosage studies were performed to assess the morphological characteristics and global transcriptional profiling to assess the effect on the transcriptional response of cell cycle and metabolism components.

Alignments with Ssd and MinD consensus sequences, and clustering analysis with Ssd and MinD proteins demonstrated that the protein encoded by *rv3660c *has similarity to *Ssd-*family proteins. Visualization of the *M. smegmatis *and *M. tuberculosis ssd *merodiploid strains and *M. tuberculosis ssd*::Tn mutant strain by scanning electron microscopy demonstrated a link between the abundance of Ssd and an elongated morphology. Bacterial filamentation is known to occur in *M. tuberculosis *and other bacteria when cell division is inhibited [7,17,18,21]. In addition, in *M. tuberculosis *visualization of the ultrastructure of the bacterial filaments reveals information about whether the inhibition is early or late in the cell division process [6,7,17,18]. When septum formation in *M. tuberculosis *is inhibited the resulting bacterial filaments are smooth and largely devoid of concentric rings indicative of established septal sites that arise when cell division is inhibited at later steps. This is an important ultrastructural distinction because inhibition of cell division at the stage of septum formation has been associated with entry into non-replicating persistence and associated with growth in macrophages [22]. Therefore, the observation that the *ssd *merodiploid strains of either *M. smegmatis *or *M. tuberculosis *displays a filamentous morphology devoid of septa is consistent with inhibition of septum formation, a characteristic associated with *in vivo *growth [22]. In addition to *rv3660c *being annotated as encoding a septum site determining protein it has also been associated experimentally with altered septum formation *via *inhibition of FtsZ polymerization and transcriptional mapping [6]. These results are fully consistent with being a putative septum site-determining protein.

Coincident with the altered growth and morphology, the *M. tuberculosis ssd *merodploid strain exhibited an adaptive genetic program that has been associated with survival and virulence. Reports of transcriptional profiles of *M. tuberculosis *exposed to a variety of conditions thought to model the *in vivo *growth environment including hypoxia, nutrient starvation, and murine infection revealed a set of common genes of the *dosR *regulon and those involved in lipid metabolism, cell wall maintenance and remodeling, and alternative respiration and redox balance [14,23-28]. When gene expression in the *M. tuberculosis ssd *merodiploid strain was evaluated, it was found that in conjunction with induction of the *dosR *regulon there was a *Dos-*like response characterized by an upregulation of genes involved in fatty acid degradation, anaerobic respiration, electron transport or redox-potential, and a down-regulation of ribosomal proteins and protein synthesis. Importantly, in the *ssd *mutant, these genes did not display a significant difference in transcriptional activity, indicating that Ssd plays a role in *Dos*-regulation and cellular adaptation under unique environmental conditions along with septum regulation.

In addition to the *Dos*-response, increased expression of *ssd *resulted in an induction of a unique alternative sigma factor response. The responsive sigma factors have been associated with adaptation to environmental stresses and virulence [29,30]. SigF has been associated with phosphate uptake, antibiotic treatment and drug tolerance [31-33]. SigG and SigH are known to be induced under stress conditions associated with DNA damage and heat and oxidative-stress responses, respectively [33,34]. SigI is directly upregulated by SigJ expression, which controls an alternative H_2_O_2 _resistance pathway for survival in the macrophage [35]. Other sigma factors such as SigL and SigM are thought to be involved in remodeling of the bacterial cell surface and production of proteins such as esat6-homologs that are necessary for survival and persistence in animal models of tuberculosis that closely mimic human infection [36,37]. Since it has been proposed that the role of these rarely expressed alternative sigma factors are related to host-specific conditions then the unique profile elicited by increased *ssd *expression demonstrates a role for Ssd in modulation of septum formation and cell division as part of the global adaptive strategy for survival in the host.

## Conclusion

In order to survive, *M*. *tuberculosis *must adapt to a stressful intracellular environment, which requires a global alternative adaptive response. Among the adaptive responses, the Dos-response is the best characterized, and has been associated with virulence. In addition to the Dos-regulon, other adaptive responses including regulation of cell division and cell cycle progression are involved in establishing a non-replicating persistent lifestyle. While all the components involved in regulation and metabolic adaptation regarding cessation of growth and non-replicating persistence in *M. tuberculosis *have yet to be defined, the results presented here substantiate Ssd as a component of a global regulatory mechanism that promotes a shift into an altered metabolic state. This is the first report providing evidence linking a regulatory element of septum formation with an adaptive response associated with virulence and non-replicating persistence in *M. tuberculosis*. Clearly, further experimentation is required to elucidate the precise mechanism of action of Ssd in regulating septum formation and its role in adaptive metabolism during stress.

## Methods

### Bioinformatic analysis

To identify putative MinD or septum site determining proteins encoded in *M. tuberculosis*, a MinD and a Ssd consensus-model sequences was created from alignments of protein sequences annotated as MinD (OMA Group 78690) or as septum site determining proteins (OMA Group 73337) from a variety of bacterial species. The resulting MinD and Ssd consensus model sequences were then used to search and identify proteins encoded in the *M. tuberculosis *genome. In all BLAST searches, the percent identity and score were optimized.

### Molecular biology and bacterial strains

The *ssd *(*rv3660c*) open reading frame was PCR amplified from *M. tuberculosis *H37Rv genomic DNA using AccuPrime pfx DNA polymerase (Invitrogen) with primer sequences 5'-ctgaccgatccgggg and 3'-gtgccatcccgccgt engineered with asymmetric NdeI and HindIII restriction sites respectively, to facilitate cloning into the extrachromosomal mycobacterial vector *p*VV16. Transformation into *M. tuberculosis *H37Rv and selection were performed as previously described [17]. For all experiments *M. tuberculosis *merodiploid and the *rv3660c *mutant strain (Tn mutant E150, provided by TBVTRM contract: HHSN266200400091c) were cultivated at 37°C in Middlebrook 7H9 liquid medium supplemented with 0.2% glycerol, 10% OADC (oleic acid, albumin, dextrose and catalase enrichment), and 0.05% Tween 80 or on supplemented Middlebrook 7H11 agar medium containing 50 μg/ml kanamycin when necessary.

### Ultrastructure analysis by scanning electron microscopy

For visualization of bacterial ultrastructure by SEM, bacterial cells were washed three times in PBS, pH 7.4, and fixed with 2.5% gluteraldehyde in Buffer A (0.1 M potassium phosphate (pH 7.4), 1 mM CaCl_2 _and 1 mM MgCl_2_) at 4°C for 24 hrs. The fixed cells were collected by centrifugation, washed three times in Buffer A and treated with 1% OsO_4 _in Buffer A for 30 minutes at 4°C. After treatment, cells were washed three times with Buffer A. and prepared for SEM with a graded series of ethanol treatments (20-100%). Ultrastructure examination was performed using a JOEL JEM -100CX electron microscope.

### Global transcriptional profiling

For transcriptional analysis, three independent biological replicates of *M. tuberculosis *H37Rv control strain, three independent biological replicates of a *M. tuberculosis *H37Rv *ssd *merodiploid strain and three independent biological replicates of a *M. tuberculosis *H37Rv *ssd*::Tn mutant strain were grown to mid-log phase growth (O.D._600 nm _= 0.3 - 0.4), harvested by centrifugation, and subjected to TRIzol before RNA isolation. Following physical disruption with 0.1 mm zirconium grinding beads, total RNA was purified using an RNeasy kit (Qiagen) as previously described [6]. Labeled cDNAs were generated using direct labeling from 5 μg of total RNA and hybridized to *M. tuberculosis *whole genome DNA microarrays obtained from the TB Vaccine Testing and Research Materials Contract (HHSN266200400091c) at Colorado State University as described [6]. Slides were scanned with a Genepix 4000B scanner. Global normalization was performed on the raw fluorescent intensities, and each feature of the array (Cy3 and Cy5) was normalized to the mean channel intensity and subjected to Anova single factor analysis. Transcriptionally active open reading frames were considered to be those with SNR >2 and a *P *value of ≤ 0.05. GEO accession # *Pending submission/data release*. Self-organizing map (SOM) analysis was performed using all transcriptionally active open reading frames.

### Quantitative real-time PCR

Quantitative real-time PCR was performed on selected open reading frames to verify transcriptional expression found by microarray as described [6]. Quantitative RT-PCR primers were designed according using Primer-3 and analyses were performed using SYBR-green chemistry (Invitrogen). RNA isolation and cDNA preparation was carried out as described above. PCR amplification was performed with a thermocycling program of 55°C for 5 min then 95°C for 2 minutes, 45 cycles of 95°C for 15 sec, 60°C for 30 sec, and 72°C for 45 sec. The relative number of transcripts for each gene was determined based on linear regression analysis of 100 ng, 10 ng, and 1 ng of *M. tuberculosis *genomic DNA. The total number of targets (n) were calculated by the equation n = a + b log (x) where "a" is the intercept and "b" is the slope of the standard curve, and "x" is the threshold cycle obtained by amplifying n targets. All reactions were performed in triplicate on at least three independent biological replicates.

*sigA *and 16S was monitored to provide additional internal controls.

## Authors' contributions

KE carried out the experimental studies and RC performed the bioinformatics. RAS designed the studies, and coordination of the manuscript. All authors participated in drafting, and editing the final manuscript. All authors have read and approved the manuscript.
